# Announcement: Fourth International Symposium on Speciation of Elements in Biological, Environmental and Toxicological Sciences (Whistler 2000 Speciation Symposium)

**DOI:** 10.1155/MBD.1999.370

**Published:** 1999


					WHISTLER 2000 SPECIATION SYMPOSIUM
Fourth International Symposium on Speciation of Elements in
Biological, Environmental and Toxicological Sciences
http://sciserv.mcmaster.ca/biochem/speciation/
June 25 July 1, 2000, WhistlerConferenceCentre,WhistlerResort,BritishCoMia, Canada
Goals and Scope
The aim of the speciation symposia is to facilitate interdisciplinary and intersector discussion about all
aspects of elements requiring an understanding of speciation, including" analytical science, geochemistry,
biochemistry, clinical chemistry, environmental science, toxicology, essentiality and nutrition, medical uses,
environmental and occupational health, and regulatory aspects. The scope of the scientific programme is
defined by the working definition of speciation endorsed in 1994 by participants of the Second Speciation
Symposium in this series" Speciation is the occurrence of an element in separate identifiable forms (i.e.,
chemical, physical, or morphological state).
The programme will comprise 4 days of oral presentations, posters, and discussion. All contributors are
asked to focus on recent research results. Oral presentations (invited or submitted) will be 20 or 30 minutes
duration. The tradition, established at previous symposia in this series, of assigning a prominent and central
role to poster presentations is to be maintained. Consequently, concurrent sessions or.ganised by themes will
be held after the formal viewing period to encourage in-depth feedback and discussion. In these special
discussion groups, each author will be allotted 5 minutes for an oral presentation of the salient features of her
or his poster.
The aim of the meeting is to link the various fields of activity mentioned above under Goals and Scope
through topics pertaining to natural or contaminant species of the elements: new developments in
methods/techniques of species determination; source characterisation and multi-media transport;
bioavailability, uptake, bioaccumulation and metabolic fate; environmental and biological monitoring;
essentiality, toxicity & resistance and associated mechanisms of action.
Instructions for the preparation and submission of abstracts are available from the web page at
http://sc serv.mcmaster.ca/biochem/spec at on/.
Michael W. Blades, Department of Chemistry, University of British Columbia, Canada. blades@chem.ubc.ca
Peter G.C. Campbell, INRS-EAU, University ofQuebec, Canada. campbell@uquebec.ca
Helen M Crews, MAFF CSL Food Science Laboratory, U.K.h.crews@csl.gov.uk
W.R. (Bill) Cullen, Department of Chemistry, University of British Columbia, Canada.. wrc@chem.ubc.ca
Harp Minhas, The Royal Society of Chemistry, Cambridge, UK. JEM@rsc.org
Evert Nieboer, McMaster University, Canada and University ofTromso, Norway. nieboere@fls.mcmaster.ca
Yngvar Thomassen, National Institute of Occupational Health, Norway. yngvar.thomassen@stami.no
.'%:. ," ,"',.:'.,...'."...'
Registration and Accommodation
Venue West Conference Services; email: congress@venuewest.com
Local Organization
Dr Michael W Blades; emaii: blades@chem.ubc.ca
Programme
Dr Evert Nieboer email: nieboere@flas.mcmaster.ca
Institutional Sponsors:
The Institute of Environment and Health, McMaster University. Hamilton. Ontario, Canada
Departnent oi" Chemistry, The University of British Colunbia, Vancouver, Canada
National Institute of Occupational Health, Oslo, Norway
Institute of Colmunity Medicine, LIniversity ofTromsm Norway
370

				

